# Mucosal Vaccination with Heterologous Viral Vectored Vaccine Targeting Subdominant SIV Accessory Antigens Strongly Inhibits Early Viral Replication

**DOI:** 10.1016/j.ebiom.2017.03.003

**Published:** 2017-03-08

**Authors:** Huanbin Xu, Anne-Marie Andersson, Emeline Ragonnaud, Ditte Boilesen, Anders Tolver, Benjamin Anderschou Holbech Jensen, James L. Blanchard, Alfredo Nicosia, Antonella Folgori, Stefano Colloca, Riccardo Cortese, Allan Randrup Thomsen, Jan Pravsgaard Christensen, Ronald S. Veazey, Peter Johannes Holst

**Affiliations:** a Tulane National Primate Research Center, Tulane University School of Medicine, Covington, LA 70433, USA; b Center for Medical Parasitology, Department of Immunology and Microbiology, University of Copenhagen, 1014, Denmark; c Department of Mathematical Sciences, University of Copenhagen, 2100, Denmark; d Department of Biology, University of Copenhagen, 2100, Denmark; e ReiThera, viale Città d'Europa 679, 00144 Rome, Italy; f CEINGE, via Gaetano Salvatore 486, 80145 Naples, Italy; g Department of Molecular Medicine and Medical Biotechnology, University of Naples Federico II, via S. Pansini 5, 80131 Naples, Italy; h KEIRES, Bäumleingasse 18, CH 4051 Basel, Switzerland; i Department of Immunology and Microbiology, University of Copenhagen, 2200, Denmark

**Keywords:** Heterologous viral vectored prime-boost immunization, Genetic adjuvant

## Abstract

Conventional HIV T cell vaccine strategies have not been successful in containing acute peak viremia, nor in providing long-term control. We immunized rhesus macaques intramuscularly and rectally using a heterologous adenovirus vectored SIV vaccine regimen encoding normally weakly immunogenic tat, vif, rev and vpr antigens fused to the MHC class II associated invariant chain. Immunizations induced broad T cell responses in all vaccinees. Following up to 10 repeated low-dose intrarectal challenges, vaccinees suppressed early viral replication (P = 0.01) and prevented the peak viremia in 5/6 animals. Despite consistently undetectable viremia in 2 out of 6 vaccinees, all animals showed evidence of infection induced immune responses indicating that infection had taken place. Vaccinees, with and without detectable viremia better preserved their rectal CD4 + T cell population and had reduced immune hyperactivation as measured by naïve T cell depletion, Ki-67 and PD-1 expression on T cells. These results indicate that vaccination towards SIV accessory antigens vaccine can provide a level of acute control of SIV replication with a suggestion of beneficial immunological consequences in infected animals of unknown long-term significance.

In conclusion, our studies demonstrate that a vaccine encoding subdominant antigens not normally associated with virus control can exert a significant impact on acute peak viremia.

## Highlights

• Mucosal heterologousvirus-vectored vaccine used with MHC class II associated invariant chain linked SIV accessory antigens
• Mucosal vaccination targeting subdominant antigens delay SIV mac251 replication in rhesus macaques.
• Longterm reduction of immune hyperactivation following SIV infection of vaccinated macaques.

Mucosal immunization is used with heterologous viral vectors and a genetic adjuvant to raise responses against poorly immunogenic SIV antigens. Following repeated low-dose challenge we observed delayed establishment of chronic phase viremia and reduced immune hyperactivation 6 months after established infection. Vaccination was found to strongly reduce viremia at early, but not late time points, after detected infection and in 2 out of 6 animals infection could only observed as virus induced T cell responses. Subdominant antigen vaccines may thus be used to delay SIV mac251 infection and can enable control of chronic viremia in a minority of cases.

## 1 Introduction

Novel vaccine strategies are needed for an effective HIV vaccine. The most successful vaccine strategies to date involved live attenuated viruses (Daniel et al. 1992), yet the potential for reversion to pathogenic viruses makes these too risky for serious consideration as vaccine candidates. Adenoviral vectors are a prime candidate to replace live attenuated vaccines, since they have a genome large enough to incorporate genes for several antigens, express antigens for extended periods of time and induce stable and protective effector memory T cells (Finn et al. 2009; Holst et al. 2008; Holst et al. 2015; Steffensen et al. 2013). Nevertheless, prior trials with adenoviral vectors have only shown partial efficacy, and in some cases, seemingly promoted infection (Buchbinder et al. 2008).

A potentially critical problem faced by both live-attenuated and non-persisting vectored immunization is immunodominance. The initial immunization selects for the most immunogenic T cell specificities, which may become highly dominant following challenge, favoring early virus escape (Liu et al. 2012). To circumvent this problem, we reasoned that antigens naturally expressed in abundance in the early stages of infection (e.g., gag) could be replaced with accessory antigens, provided that stronger and broader responses could be elicited towards these less immunogenic antigens. In mice, such an experiment resulted in broader immune control against a persistent lymphocytic choriomeningitis virus (LCMV), as compared to the response obtained using only the most immunogenic antigen (Holst et al., 2015). Against HIV, such a strategy of avoiding the most dominating antigens offers the additional benefit of targeting epitopes that has not been evolutionary modified for immune escape (Monaco et al. 2016). For targeting SIV, we therefore constructed two different adenoviral vectors, human adenovirus type 5 vector and chimpanzee type 63 adenoviral vector (ChAd63), expressing accessory antigens not classically associated with strong immune responses to this infection (tat, vif, rev and vpr). To overcome the weak intrinsic immunogenecity of the selected antigens, we used our previously published MHC class II associated invariant chain based genetic adjuvant (Capone et al. 2014; Holst et al., 2008; Holst et al., 2015; Spencer et al. 2014) coupled to SIV mac239 derived tat, vif, rev and vpr expressed as a single fusion protein, and administered these vaccines in a combined rectal and intramuscular heterologous prime-boost immunization. We have previously found combined mucosal and parenteral immunization to be critical for optimal for mucosal immunosurveillance, effector cell mobilization and control of acute and chronic infection (Hoegh-Petersen et al. 2009; Uddback et al. 2016). We first ascertained that these vectors were immunogenic in mice. We then vaccinated 6 Indian origin rhesus macaques three months apart to assess whether non-classical epitopes could induce control of pathogenic SIVmac251 challenge as compared to 6 unimmunized controls.

Here we show that all animals showed broad vaccine induced CD8 + T cell responses and a trend towards delayed or absent viremia following repeated low-dose intra-rectal challenges (P = 0.08). After 10 rectal challenges, all 6 controls were infected whereas 2 vaccinated animals remained aviremic. Furthermore, 3 out of the 4 infected vaccinees demonstrated a markedly attenuated early infection taking several weeks to reach an otherwise normal set-point viremia. Despite undetectable viremia in two animals, all vaccinees exhibited infection induced T cell responses demonstrating that all animals had become infected during the challenges, with two animals achieving rapid and durable viremic suppression. Consequently, we have observed a rather pronounced vaccine induced effect on early viral replication (P = 0.01 for reduced early virus load). All vaccinated animals with or without directly detectable infection exhibited long term immunological benefits such as reduced rectal CD4 + T cell depletion and highly limited CD8 + T cell hyperactivation.

Our results demonstrate that SIV accessory antigens vaccine can profoundly improve acute virological control of SIV mac251 challenge, with potential long-term immunological benefits.

## 2 Materials & Methods

### 2.1 Animals

CD1 mice were purchased from Taconic M&B (Ry, Denmark). The murine immunization studies were approved by the Danish National animal experiments inspectorate. For the nonhuman primate studies, purpose bred, Indian-origin rhesus macaques were obtained from, and housed at the Tulane National Primate Research Center (Tulane). Animals were randomly assigned to treatment or control groups before MHC typing was performed. All procedures were carried out in strict accordance with the recommendations in the Guide for the Care and Use of Laboratory Animals of the National Institutes of Health (NIH) and with the recommendations of the Weatherall report: “The use of non-human primates in research”. The Institutional Animal Care and Use Committee (IACUC) of Tulane University approved all macaque procedures described under protocol permit number P0181. All procedures were performed under anesthesia using ketamine or telazol, and all efforts were made to minimize stress.

### 2.2 Vaccines and Antigen Design

To induce potent CD8 + T cell responses from otherwise weak antigens we applied the genetic adjuvant MHC class II associated invariant chain (Ii) and fused this molecule to a tat, vif, rev and vpr fusion antigen with sequences from mac239 (tvrv). The tat antigen contained reported inactivating mutations in the cysteine position 56 (C56S) and arginine in position 82 (R82L) corresponding to the C27S and R55L mutations described by Mayol et al. for HIV tat (Mayol et al., 2007). Ii functions as a potent genetic adjuvant for CD8 + T cells (Capone et al., 2014; Holst et al., 2008; Spencer et al., 2014). The antigen was encoded in heterologous adenovirus vectors based on chimpanzee adenovirus type 63 and human type 5 (Colloca et al., 2012). The hAd5 vector incorporated the rhesus macaque Ii isoform 2 sequence amino acids 1–190 whereas the chimpanzee type 63 adenoviral vector (chAd63) incorporated the human Ii isoform 1 sequence as genetic adjuvants (Capone et al., 2014). The viruses were rescued by co-transfection in HEK293 cells and cloned by agarose overlay (hAd5) or as full-length vector genomes in BJ5183 cells (Ch63) before rescue on adenovirus producer cells. Following rescue the viruses were amplified using standard methods and purified using CsCl banding after ultracentrifugation (Becker et al. 1994). Adenovirus particle titers were determined by OD measurements at 260 nm and infectivity of hAd5 vectors was verified by Adeno-X rapid titer kit. The integrity of the adenovirus genomes was determined using restriction enzyme digest of purified vector genomes and direct sequencing of the antigen expression cassette.

### 2.3 Antigen Expression

To verify expression of the antigen HEK293 cells were infected with hAd5-tvrv, Ch63-tvrv, hAd5 control or Ch63 control and 48 h post infection cell lysate was used for western blotting. The primary detection reagent was mac251 specific polyclonal antisera obtained through the NIH AIDS Reagent Program, Division of AIDS, NIAID. Polyclonal Rabbit anti-human HRP conjugated antibody (Dako P0214) was used as the secondary antibody with LumiGLO® Chemiluminescent Substrate System (KPL 54-61-00) as detection reagent and the blot was read using an ImageQuant LAS 4000 biomolecular imager.

### 2.4 Mouse Immunizations and T Cell Responses

Mice were immunized subcutaneously behind the footpad of the right hind leg using 2 × 10^7^ infectious units of hAd5 and 10^9^ particles of Ch63 vaccine. For measurements of CD8 + T cell specific immune response, single cell suspensions of splenocytes were obtained by pressing the organs through a fine steel mesh, followed by centrifugation and resuspension in RPMI cell culture media. The cells were then incubated with overlapping peptide pools from the vif and vpr proteins obtained from the NIH AIDS Reagent Program at a concentration of 1 μg/ml of each peptide. Stimulation and staining was performed as described (Christensen et al. 2003) except that the cells were incubated without monensin for the first hour and then for 5 h in the presence of 3 μM of monensin. Functional epitope specific CD8 + T cell responses were enumerated by surface staining for CD8 (Pe/Cy5.5 or Pacific Blue), CD44 (APC/Cy7), CD19 or B220 (PerCP/Cy5.5 and pacific blue respectively) and intracellular staining for IFN-γ (APC). Thus, cells enumerated in this study represent numbers of CD8 +, CD44 +, IFN-γ + and CD19/B220- cells in the spleens of analyzed mice and are presented after subtraction of background responses seen without peptide stimulation. Total numbers were calculated by multiplying the total number of cells in the spleens determined using a hemocytometer, and the percentage of specifically gated cells. All antibodies were mouse cells were purchased from Biolegend. Cell samples were run on a Becton-Dickinson LSRII FACS machine, and data analyses were performed using Flow Jo (Tree Star) software.

### 2.5 Primate Immunizations and Challenges

To evaluate the efficacy of the vaccine 6 Indian origin rhesus macaques were vaccinated with hAd5 vectors encoding the tat, vif, rev, and vpr antigen and boosted 3 months later with Ch63 vectors. First, macaques (n = 6) were intramuscularly and rectally inoculated with 5 × 10^10^ particles of the hAd5 vectored DNA vaccine in 1 ml inocula at week 0, and then boosted intramuscularly and rectally with 2 × 10^10^ particles of the Chimp Ad63 vector vaccine in 1 ml inocula 12 weeks later (wk 12). An additional 6 macaques were sham inoculated with PBS as controls. Following the initial animal experimentation protocol; beginning 4½ months after the last immunization, macaques were rectally challenged weekly for 5 weeks with 1 ml of a 1:500 dilution of SIVmac251 made by Ronald C. Desrosiers (New England National Primate Research Center) and provided by Nancy Miller (NIAID, NIH) (Tuero et al. 2015). Viremia was assessed on samples collected after each challenge, but analyzed after the completion of all the challenges. Since two sham controls and three vaccinees remained uninfected, a new infection protocol was made and an additional 5 weekly rectal challenges were resumed beginning at wk. 43, but this time with 1 ml of a 5 fold higher (1:100) dilution. Blood was collected weekly throughout challenges and for 4 weeks after the last challenge before virus load analysis. Monitoring was continued with monthly samples thereafter for flow cytometry and monitoring plasma viremia. Plasma viral load was detected with a PCR assay with a linear detection limit of 28 copies/ml of plasma as previously described (Monjure et al. 2014). Lymph node biopsies were collected from all animals before the first challenge and 6 months after the last challenge. Cell suspensions prepared from lymph nodes were stained by CD3, CD4 and CD8, and CD4 + T cells were sorted/quantified from lymph nodes by FACS Aria for RT-QPCR using an ultrasensitive protocol with a detection limit < 2 copies.

### 2.6 Primate Antigen Specific T Cell Responses

To detect SIV-specific T cell responses in macaques, PBMCs (1 × 10^6^) isolated from heparinized blood were incubated at 37 °C in a 5% CO_2_ environment for 6 h in the presence of RPMI 1640-10% FCS alone (unstimulated), a pool of 15-mer Gag, Tat, vif, Vpr or Rev peptides (5 μg/ml each peptide), or staphylococcal enterotoxin B (1 μg/ml; Sigma-Aldrich, St. Louis, MO, USA) as a positive control. All cultures contained brefeldin A (Sigma-Aldrich), as well as 1 μg/ml of anti-CD49d and anti-CD28 costimulatory molecules (BD Biosciences). Cultured cells were stained with monoclonal antibodies specific for CD3 (SP34), CD4 (L200), CD8 (SK1), and Aqua Live/Dead (Invitrogen). After being fixed and permeabilized with Cytofix/Cytoperm solution (BD Biosciences), cells were stained with antibodies specific for IFN-γ (4S.B3) and TNF-α (MAB11) and washed by Perm/wash buffer (BD Biosciences). All antibodies and reagents were purchased from BD Biosciences PharMingen (San Diego, CA, USA). Labeled cells were finally resuspended in BD Stabilizing Fixative Buffer, and acquired on a FACSVerse cytometer (Becton Dickinson, San Jose, CA, USA). Data were analyzed using FlowJo software (Tree Star). Cut-off values for positive responses were based on responses in unvaccinated animals before challenges and without antigen stimulation. The cut-off value was 0.32% of IFN-γ positive CD8 + T cells and 0.06% of IFN-γ positive CD4 + T cells. ELISA assay: MaxiSorp (NUNC) flat bottom plates were coated with 0,1 μg Tat protein (obtained from NIBSC. Cat. No. ARP685) in carbonate buffer overnight and subsequently blocked with PBS (5% BSA, 0.05% Tween). Monkey serum samples were diluted 1:200 in PBS (5% BSA, 0.05% Tween), 2-fold dilution series were made and 100 μl was added to each well and incubated for 1 h. The secondary antibody was HRP-linked polyclonal rabbit anti-human (HRP-coupled, po no. P0214) diluted 1:2000 and 100 μl was added to each well. Antibodies were detected by adding 100 μl OPD buffer for 20 min, and the absorbance at 490 nm was measured on VERSAmax microplate reader. Washing with PBS (0,1% Tween) was performed between each step.

### 2.7 Tissue Collection and Immune Cell Phenotyping

Flow cytometry for surface staining was performed using standard protocols (Xu et al. 2015). Cells from lymph nodes and blood were stained with: CD3 (SP34), CD4 (SK3), CD8 (SK1), CD95 (DX2), CD45RA (L48) and CD28 (CD28.2) (all from BD Biosciences Pharmingen, San Diego, CA), CXCR5 (MU5UBEE, eBioscience), PD-1 (EH12.2H7, BioLegend), CCR7 (GO43H7) and LIVE/DEAD Fixable Aqua Dead Cell Stain Kit (Invitrogen, Grand Island, NY). For assessing proliferation, PBMC was surface stained, treated with FACS lysing solution, washed, and intracellularly stained with anti-Ki67 (Clone B56). Isotype-matched controls were included in all experiments. Samples were resuspended in BD Stabilizing Fixative (BD Biosciences) and acquired on a FACS FORTESSA (Becton Dickinson, San Jose, CA). Data were analyzed with Flowjo software (Tree Star, Ashland, OR).

### 2.8 Statistics

Various non-parametric tests were applied to compare the number of escaped challenges between vaccinated and unvaccinated animals: we used a Kaplan-Meier plot and a log-rank test to compare the survival function for the number of escaped challenges, and a Fisher's exact test was used to compare the frequency of escaping 0–2, 3–4, or > 5 challenges across treatment groups. Both methods provide valid test results since we used randomization-based exact p-values as recommended by Nolen et al. (2015) (Nolen et al. 2015).

Early mean virus load 1–4 weeks after successful challenge were compared using Mann-Whitney *U* test. Two animals which never had detectable infection were included in this analysis using the assay detection limit. This was justified by the detection of immune responses to non-vaccine encoded SIV proteins after the fifth challenge.

To examine the likelihood of a random and independent vaccine effect on detectable acquisition and viral replication we used a non-parametric test based on the number of escaped challenges and the mean viral load in the first 4 weeks after infection (censored if no infection occurred). Formally, we added the Mann Whitney *U* test statistics for (i) comparing the number of escaped challenges, and (ii) for comparing the viral load across groups where only infected animals were included in the latter. The exact distribution of the combined test and the two-sided p-value was approximated using a Monte Carlo sampling approach, with data simulated under the relevant null hypothesis that neither detectable acquisitation nor early viral replication was affected by vaccination.

Differences in T cell responses and phenotype distribution between vaccinees and controls were compared using Mann Whitney *U* test. Within group differences over time were compared using Wilcoxon sign test. Differences between groups of changes over time were analyzed by comparing the grouped individual animal changes using Mann-Whitney *U* test.

All statistical analyses were carried out using R [R Core Team (2014). R: A language and environment for statistical computing. R Foundation for Statistical Computing, Vienna, Austria. http://www.R-project.org/]. For non-parametric tests we used the R-packages ‘exactRankTest’ and ‘coin’ for computation of exact p-values.

## 3 Results

### 3.1 The Ii-tvrv Vaccine is Expressed, and Elicits Responses in Mice

**Fig. 1:**
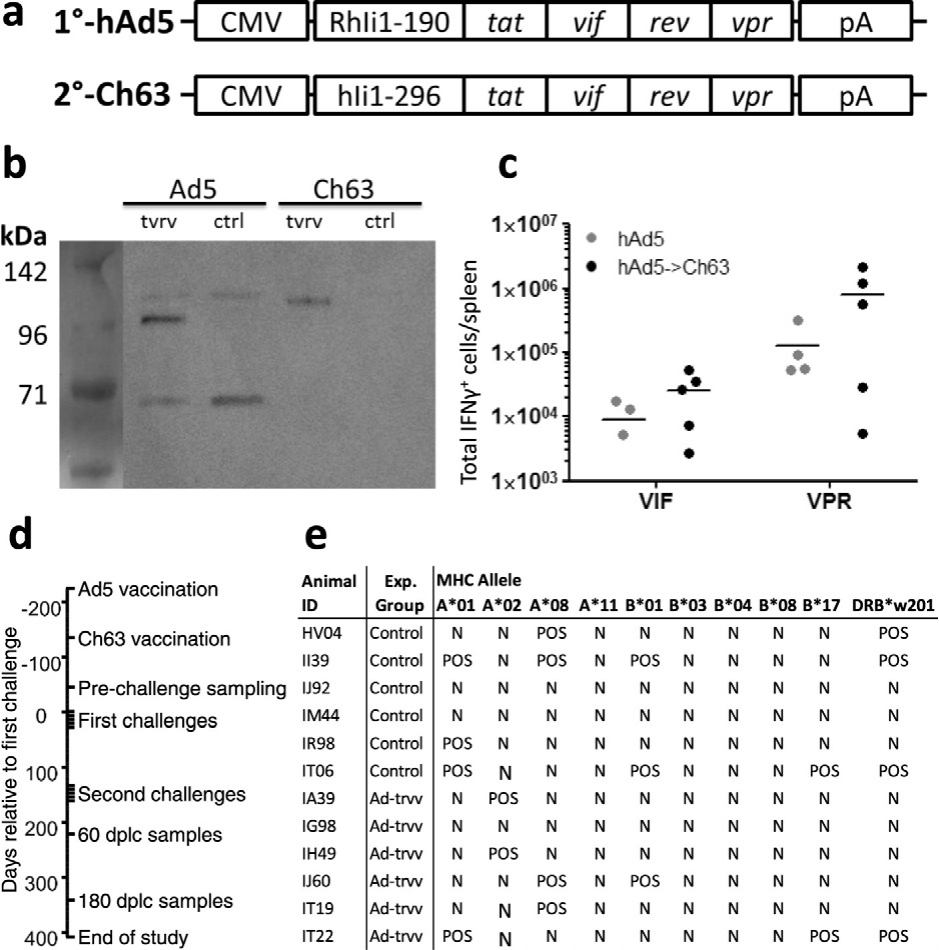
Design, expression and in vivo immunogenicity of adenoviral vaccines and primate trial set-up. a, Design of the expression cassette encoded in the adenoviral vaccines. 1° vaccine depicts the hAd5 vaccine which uses Rhesus macaque MHC class II associated invariant chain amino acids 1–197 as an adjuvant for a tat, vif, rev and vpr fusion protein (tvrv) encoded under the CMV promoter and SV40 polyadenylation sequence. 2° vaccine depicts the Ch63 based booster vaccine which uses the human MHC class II associated invariant chain isoform 1 and a bovine growth hormone polyadenylation sequence, but otherwise is designed as the priming vaccine. b, the hAd5 and Ch63 vaccines and controls expressing irrelevant antigen were used to infect HEK293 cells and cell lysate were used for western blot using primate SIV infected serum and cross-reactive anti-human HRP antibody for detection. c, outbred CD1 mice were vaccinated with the hAd5 vaccine and left for 64 days (gray circles) or boosted with Ch63 vaccine after 56 days and sacrificed 8 days later (black symbols). Shown are intracellular levels of IFNγ in splenocytes from individual mice stimulated ex vivo with overlapping vif and vpr peptide pools. d, the time course of vaccinations, challenge rounds and time points for parallel samples analyzed. Dplc is an abbreviation of days post last challenge. e, all animals included in the experiment were typed for common Mamu-A and Mamu-B alleles and the DRB*203 allele after their group designation. POS denotes an animal positive for the indicated allele, N denotes negative.

The vaccine was constructed as a fusion of the MHC class II associated invariant chain to a tat, rev, vif and vpr fusion antigen. To assess the functionality and immunogenicity of the adenovirus constructs (Fig. 1a), we first confirmed expression of the transgene (Fig. 1b). Then, outbred CD1 mice were vaccinated in a heterologous prime-boost regimen initiated with human adenovirus type 5 and either boosted or not with Chimpanzee adenovirus vectors 56 days later. The antigen specific responses in these outbred mice were variable, but the results demonstrated that the antigen was immunogenic and responses were increased by the Ch63 booster immunization (Fig. 1c).

### 3.2 Ii-tvrv is Partially Effective in Controlling an SIV mac251 Challenge

**Fig. 2:**
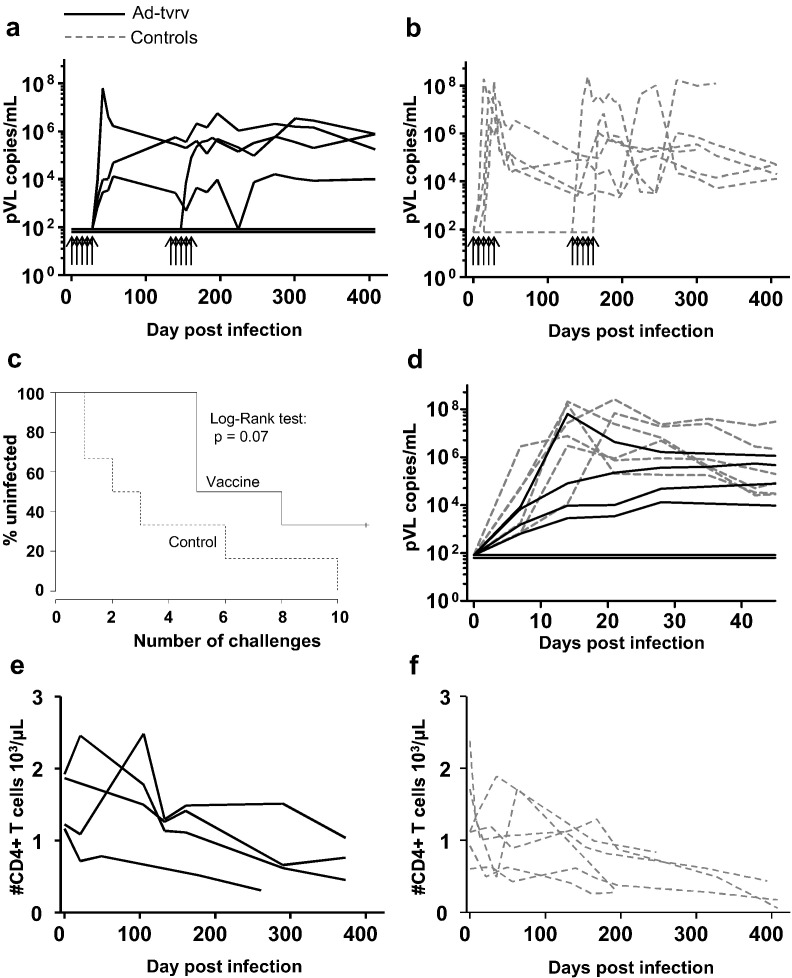
Virus acquisition, progression of the infection and CD4 + T cell decline in vaccinated animals and controls. a–b, plasma viral load in blood samples from individual macaques in vaccinated (a) and PBS injected controls (b). Arrows indicate the timing of the rectal challenges. c, detection of infection in vaccinated and controls animals. Infections were assumed to occur the week before virus was first detectable in plasma. d, progression of infection following acquisition. Shown are plasma viral load in all animals synchronized to the day of infection. Aviremic vaccinees are shown at the detection limit of the assay. e–f, CD4 + T cells in blood synchronized to the day of the successful infection in vaccinated animals (e) and controls (f).

We next included 12 Indian origin rhesus macaques in a vaccine trial, 6 sham immunized and 6 that were vaccinated with the hAd5 vector and the Ch63 vector 3 months later, followed by sampling for blood CD8 + T cell responses 3 months later again. Weekly intrarectal challenges were performed in two rounds of 5 challenges initiated 4½ month after the last immunization (immunization and challenge schedule is outlined in Fig. 1d). The macaques were randomly selected and assigned, but later typed for common MHC alleles (Fig. 1e). The known resistance alleles A*08 and B*17 were equally distributed among vaccinees and controls whereas three controls had A*01 alleles associated with prominent early gag specific T cell responses compared to one in the vaccinees. After the initially planned five low-dose challenges (1:500 dilution, see M&M) we could not detect viremia in three of the vaccinees and two unvaccinated controls. We therefore performed a second challenge round with five inoculations of a higher dose of virus (1:100): 2 vaccinated animals remained free from detectable viremia whereas the last controls became infected (Fig. 2a–c). Overall, vaccination reduced the per-exposure risk of detected infection by 64% and increased the number of challenges needed for detection of infection, but the reduced detection rate was not significant (P = 0.08). The two vaccinees that remained uninfected were also negative for viral RNA and DNA in lymph node biopsies taken 6 months after their last challenge using ultra-sensitive PCR on sorted lymph node CD4 + T cells (not shown).

Those animals in which we could directly detect infection also had a markedly different course of infection compared to the controls. Whereas all controls reached peak plasma viremia within 1 or 2 weeks after detection of first viremia, in 3 out of the 4 vaccinated infected animals, viral loads did not “peak”, but slowly reached their plateau phase four or more weeks after infection (Fig. 2d). However, once the infected vaccinees reached their plateau phase, no difference was observed in the level of viremia in vaccinated animals and controls.

As the vaccine regimen was intended as a T cell based vaccine, the apparent inability to infect 2 vaccinees and the trend towards delayed detection of acquisition was a considerable surprise. In light of the unusual absence of peak viremia and the delayed set-point viremia in 3 out of 4 animals we therefore decided to initially compare differences in viral load both based on the assumption that all animals had become infected - with some simply being able to durably control the infection below the detection limit (P = 0.01 for reduction of acute mean viral load of the first 4 positive samples, Mann-Whitney *U* test). We also tested the likelihood that the statistical insignificant delayed detection of acquisition as well as the reduction of acute viremia in 3 out of 4 animals were random and independent phenomena using a mixed test on acquisition and early viral load (P = 0.02) (cf. Materials and Methods section for a full description of the viral load analysis).

Early partial control of infection was also reflected in measurements of blood CD4 + T cells. With one exception, vaccinated animals maintained CD4 + T cells in the blood early after infection, but following the first 100 days a slow decline was observed (Fig. 2e). Not surprisingly, the 2 animals with durable viremic suppression did not exhibit any CD4 + T cell decline (not show). Unvaccinated controls exhibited a rapid reduction of CD4 + T cells within the first few weeks of acquisition of infection (Fig. 2f). When the study was ended (one year after the first challenge) two of the controls, but none of the vaccinees had been euthanized due to disease progression.

Collectively, these data shows that the vaccine reduce early SIV251 replication.

### 3.3 Antigen Specific CD8 + T Cell Responses to Vaccination and Infection

To study vaccine induced T cell responses PBMC's were collected and analyzed by peptide stimulation and intracellular staining (ICS) 3 months after the booster immunization and one, two and three weeks after detection of infection using peptide pools overlapping the sequence of the vaccine antigens tat, vif, rev, and vpr, as well as gag.

**Fig. 3:**
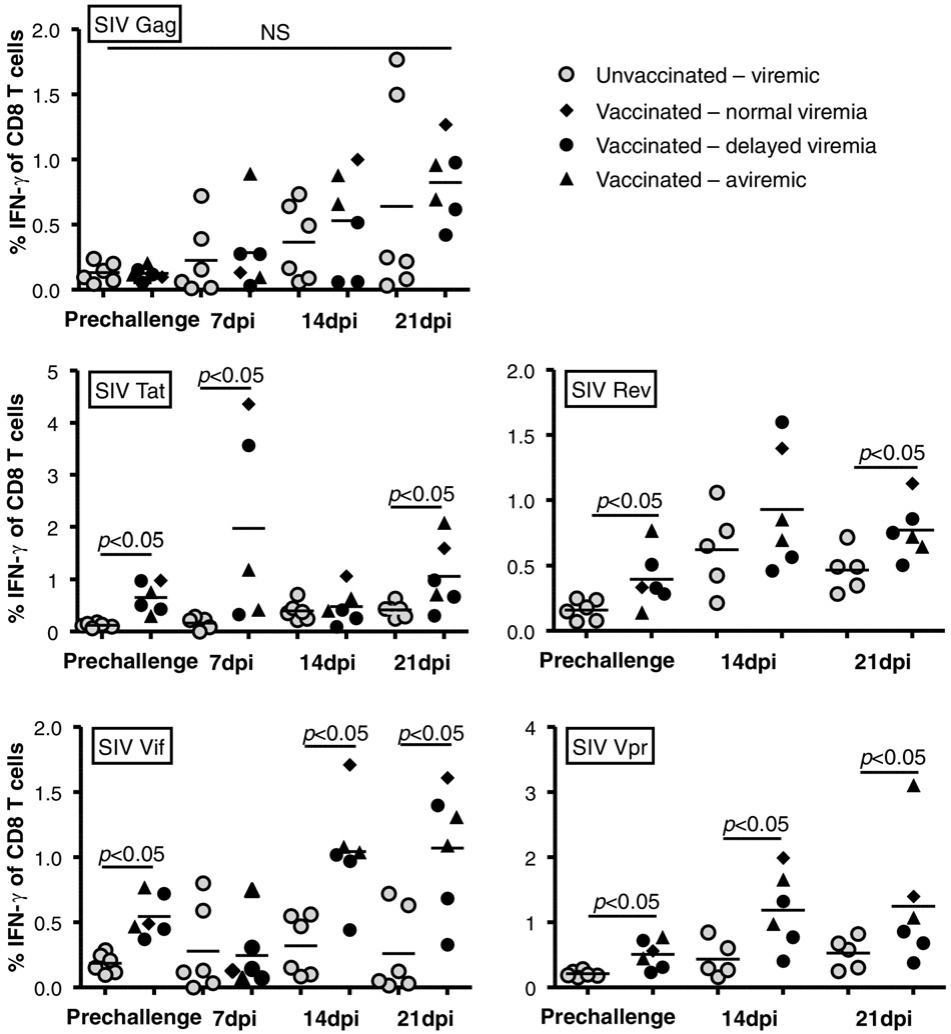
Pre- and post-exposure antigen specific CD8 + T cell responses in vaccinated animals and controls. Plots depict the percentage of gated IFN-γ + cells out of total CD8 + T cells in PBMC's from individual animals stimulated with peptide pools covering the antigens indicated in the top left corner of the plots. Values are shown for blood samples collected before challenge (cf. Fig. 1d) and at the indicated time after successful acquisition of infection. Dpi = days post infection. Vaccinated animals are grouped as indicated based on their levels of acute viremia.

In the pre-challenge samples, significant IFN-γ + CD8 + T cell responses were detected against all four vaccine encoded antigens (Fig. 3). Notably, each of the vaccinees responded to 3 or 4 of the 4 vaccine antigen peptide pools as defined by a response above the background of the assay and all of the non-immunized controls. For visualizing post exposure responses we grouped the vaccinees into the two animals that never had detectable infection (vaccinated – aviremic), the three animals that exhibited significant delays in time to reach peak viral load (vaccinated – delayed viremia) and the one animal that showed normal viral load kinetics (vaccinated – normal viremia). For the aviremic animals comparison samples were selected to correspond to the day 7, 14 and 21 dpi samples from three of the 4 initially infected and viremic vaccinees. In this analysis, it is apparent that all animals showed anamnestic responses towards the vaccine antigens, and also showed potent gag specific responses by 14 and 21 dpi (Fig. 3). This included the two animals fully protected from viremia after all 10 challenges which strongly supports the hypothesis that these animals had exhibited various levels of control of the infection rather than prevention.

### 3.4 Antigen Specific CD4 + T Cell Responses to Vaccination and Infection

**Fig. 4:**
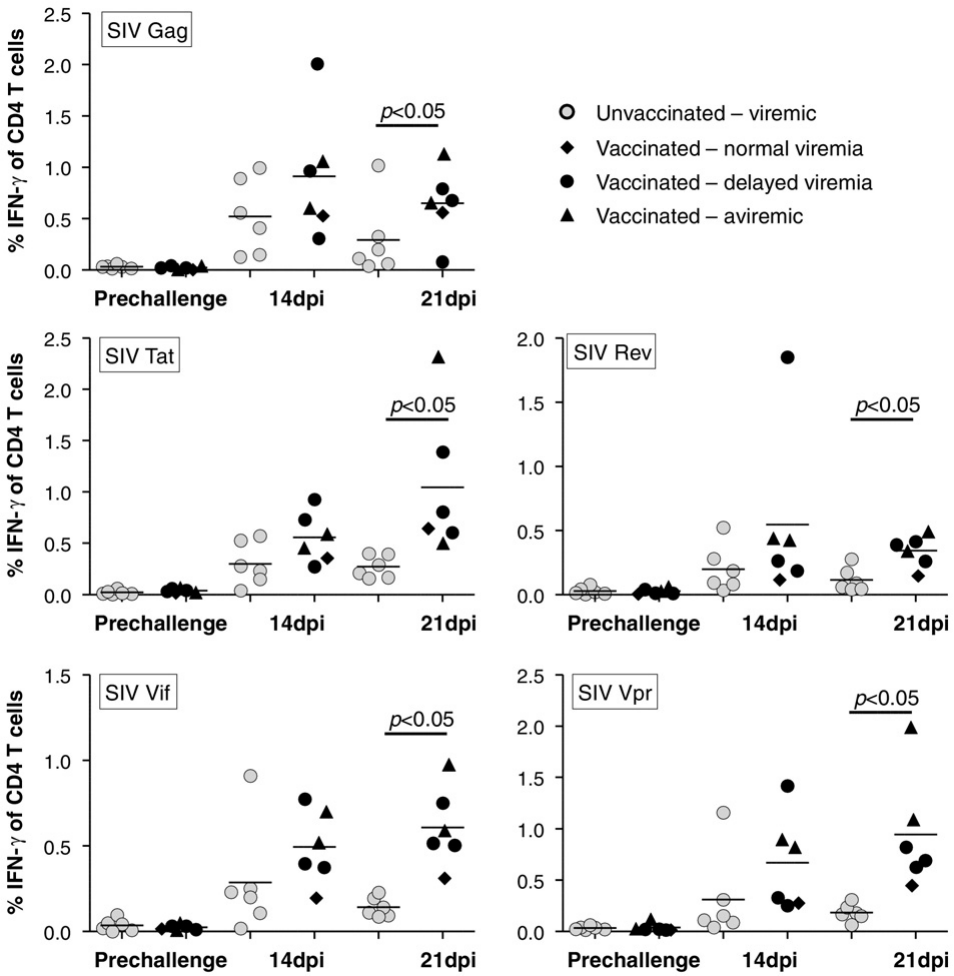
Pre- and post-exposure antigen specific CD4 + T cell responses in vaccinated animals and controls. Plots depict percentage of gated IFN-γ^+^ cells of total CD4^+^ T cells in PBMC's stimulated with peptide pools covering the antigens indicated in the top left corner of the plots. Values are shown for blood samples collected before challenge (cf. Fig. 1d) and at the indicated time after successful acquisition of infection. Dpi = days post infection. Vaccinated animals are grouped as indicated based on their levels of acute viremia.

To measure T helper cell responses, antigen specific CD4 + T cells were detected in PBMC's. Samples for these analyses were obtained 3 months post booster vaccination and 2 and 3 weeks post acquisition of infection, after peptide stimulation under conditions similar to the analysis performed on CD8 + T cells (Fig. 4). However, unlike CD8 + T cell responses, CD4 + T cell responses were not readily detectable before challenge. After challenge, vaccinated animals trended towards stronger early responses than the controls, and maintained responses from two to three weeks post infection. By three weeks post infection, the vaccinees had significantly stronger responses against all tested antigens.

### 3.5 A Role for tat Specific Antibodies?

As the vaccine included tat antigen that has been claimed to be protective via antibody mediated mechanisms (Bachler et al. 2013; Rezza et al. 2005), we also measured tat responses by ELISA against full-length tat protein (Table 1). Only the vaccinee with normal viremia demonstrated a detectable response towards tat prior to the challenges and the infected vaccinee that remained without a detectable tat response had the lowest viral load of all infected animals. Thus, tat specific antibodies seem unlikely to contribute to the delayed or suppressed viremia observed in this trial.

### 3.6 Ii-tvrv Vaccine Reduces Naïve T Cell Depletion

**Fig. 5:**
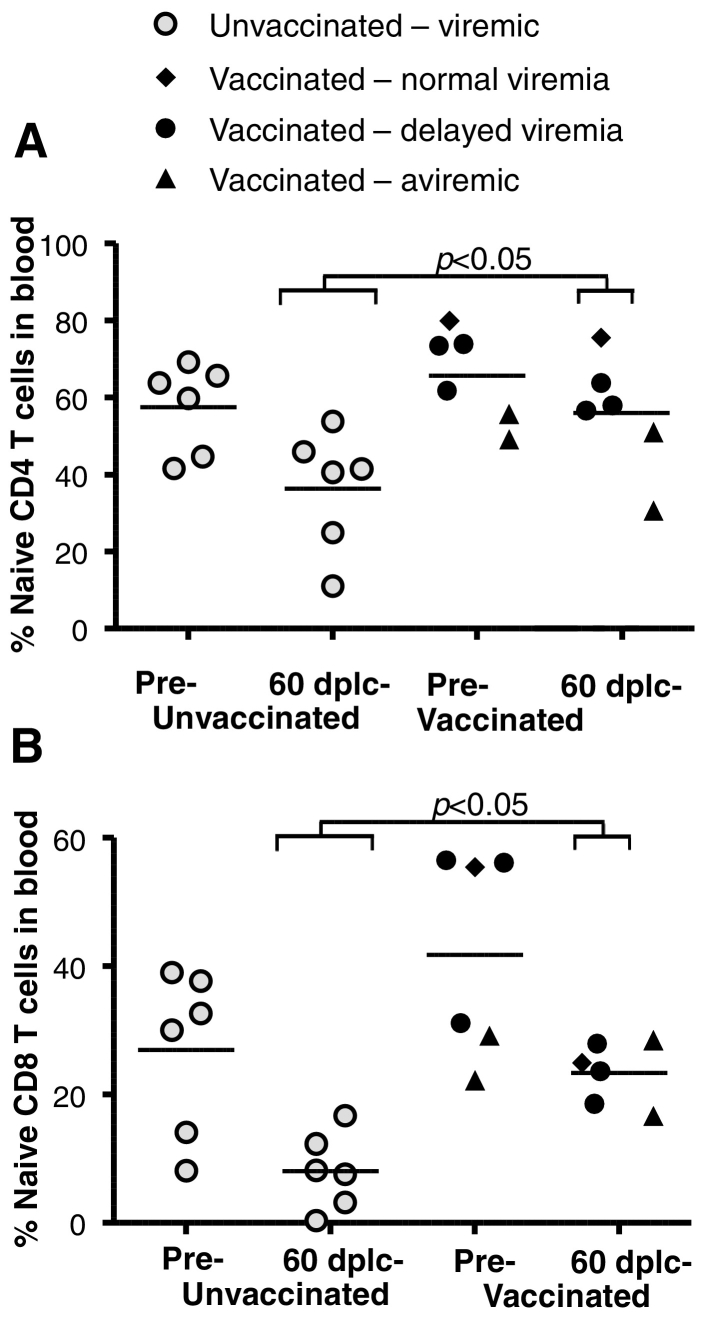
Vaccination reduces naïve T cell depletion following infection. a, naïve CD4 + T cells were enumerated based on negative staining for CD95- and positive staining for CD28 in pre-challenge (cf. Fig. 2a) and 60 dplc (days post last challenge, cf. Fig. 2a). b, naïve CD8 + T cells were enumerated based on positive staining for CD45RA and CCR7 in pre-challenge and 60 dplc blood samples. Shown are individual animals with brackets to indicate that only infected vaccinees are included in the statistical comparisons. Vaccinated animals are grouped as indicated based on their levels of acute viremia.

To explore if the improved early virus control generated by the vaccines might have had beneficial effects beyond the early reduction of viremia, we initially determined the numbers of naïve CD4 + and CD8 + T cells in PBMCs before challenge and at the relatively early time-point of 60 days post last challenge (Fig. 5). Depletion of naïve T cells was considered an informative read-out as Nishimura et al. found this depletion to predict disease activity and progression to AIDS after SIV infection (Nishimura et al. 2007) and Hazenberg et al. reported similar findings in HIV infected patients (Hazenberg et al. 2003). There were no significant differences before challenge, but after challenge and infection, we found the naïve populations to be significantly lower in the controls compared to the infected vaccinees for both CD4 + T cells (Fig. 5a) and CD8 + T cells (Fig. 5b).

### 3.7 Long-term Cellular Consequences of SIV Challenge in tvrv Infected Animals

**Fig. 6:**
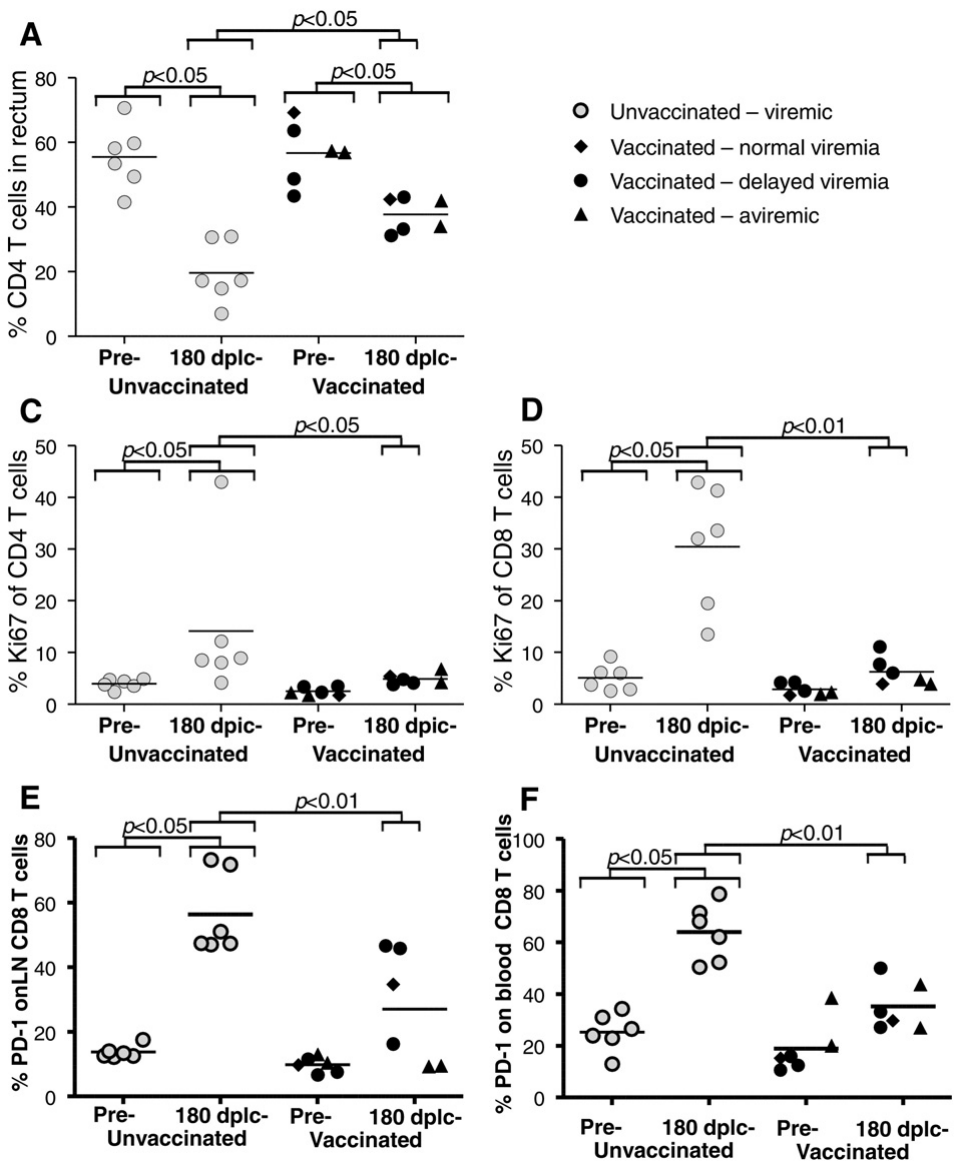
Vaccination preserved mucosal CD4 + T cells, reduces immune hyperactivation and exhaustion induced by SIV infection. Immune cell phenotyping was performed on blood and lymph node biopsies pre-challenge and 180 dplc (days post last challenge, cf. Fig. 1d). a, Percentages of CD4 + T cells from rectal biopsies were determined from total gated CD3 + T cells. b, Percentage of Ki-67 + CD4 + T cells or **c,** CD8 + T cells from PBMC's. d, Percentage of CD8 + T cells from lymph node biopsies co-expressing PD1. e, Percentage of CD8 + T cells in PBMC's co-expressing PD-1. Statistical comparisons are indicated by brackets with the width of the brackets indicating if aviremic vaccinees are included in the comparison or not. Vaccinated animals are grouped as indicated based on their levels of acute viremia.

To investigate if the improved acute viremic control would have immunological long term consequences, we undertook a series of analyses on samples taken 180 days after the last challenge, when virus loads were similar in the controls and the 4 infected vaccinees. Biopsies were used to quantitate CD4 + T cell frequencies of total rectal T cells (CD3 +). We found that both groups showed reductions in rectal CD4 + T cells (P < 0.05, Wilcoxon Signed Rank Test on all animals), including the animals without detectable infection, but the relative level of depletion was more pronounced in the controls (P < 0.05). In consequence, at 180 days post infection, the rectal CD4 + T cell counts were more depleted in controls than in infected vaccinees (P < 0.05) and all vaccinees maintained rectal CD4 + T cell frequencies of approximately 40% (Fig. 6a). This is in contrast to the systemic CD4 + T cell counts which were reduced in viremic animals. As the two vaccinated animals with an undetectable viral load have exhibited both infection induced immune responses and moderately reduced rectal CD4 + T cell counts at levels similar to other vaccinees, we have decided to comment and perform the statistical tests on the following additional results including either all vaccinees and/or excluding the two aviremic vaccinees as indicated.

### 3.8 Tvrv Vaccine Reduces Chronic Immune Activation and Immune Exhaustion

One of the important long-term consequences of HIV and SIV infection is chronic immune activation. To directly quantitate general T cell activation, we measured the proportion of CD4 and CD8 + T cells expressing the marker of recent proliferation, Ki-67. Ki-67 expression were at low levels on both CD4 + and CD8 + T cells before challenge, but in both controls and vaccinees we observed increases in the frequency of both CD4 + (Fig. 6c, significant for vaccinees only) and CD8 + T cells expressing Ki-67 (Fig. 6d). Particularly within the CD8 + T cell compartment, these differences clearly distinguished the controls from all vaccinees - also when only considering the detectably infected vaccinees that at this time point had viral loads similar to the controls (Fig. 6d). Notably, some studies have suggested that adenovirus vaccination causes generalized CD4 + T cell activation (Bukh et al. 2014). We observed no such effect, nor a trend before the challenges.

A now classical consequence of the increased immune activation and chronic high viral load in HIV and SIV infection is CD8 + T cell exhaustion. CD8 + T cell exhaustion and chronic activation correlates with the expression of PD-1 on the surface of CD8 + T cells. As anticipated, the infected control animals had profoundly increased PD-1 expression on CD8 + T cells after the challenges in lymph node biopsies (Fig. 6e), and this was also seen in detectably infected vaccinees, but significantly higher PD-1 expression levels were reached in the controls than in the viremic vaccinees (P < 0.01). As expected, the aviremic vaccinees displayed very low PD-1 expression on the CD8 + T cells from lymph node biopsies. A comparison of CD8 + T cells from PBMC samples taken before challenge and 6 months post last challenge yielded the same trends with higher PD-1 expression on controls compared to infected vaccinees (Fig. 6f).

In summary, the analyses of naïve CD4 + and CD8 + T cell depletion, the preservation of rectal CD4 + T cells, and the reduced T cell proliferation and PD-1 expression on CD8 + T cells, collectively provide a strong case that vaccination improved early cell mediated control and reduced and/or delayed SIV associated immune hyperactivation.

## 4 Discussion

The non-structural antigens tat and rev are expressed early in the virus life-cycle and together with vif and vpr, they are among the least immunogenic antigens during natural infection with HIV (Betts et al. 2002). This suggests that vaccine induced tat, vif, rev and vpr specific responses would not dominate other T cell responses after infection (Holst et al., 2015). To compensate for the expected low immunogenicity, we used the potent genetic adjuvant, the MHC class II associated invariant chain (Capone et al., 2014; Holst et al., 2008; Holst et al. 2011; Holst et al., 2015; Spencer et al., 2014), to induce robust T-cell responses prior to challenge with pathogenic SIV mac251. We decided to perform vaccinations with combined intramuscular and rectal delivery for the heterologous virus vectored prime-boost regimen in this study as this had been shown to improve mucosal immune surveillance, effector cell recruitment and long-term control of chronic infection in mouse studies (Hoegh-Petersen et al., 2009; Uddback et al., 2016). Importantly, we verified robust and broad CD8 + T cell responses against the vaccine antigens in blood (Fig. 3), and in agreement with our previous results, we observed minimal antigen specific CD4 + T cell activation (Fig. 4), and no generalized increase in T cell activation before the challenges despite the use of hAd5 vectors (Fig. 6c–d). Following repeated low-dose SIV mac251 challenges, a clear effect was seen on early virus replication observed as delayed set-point viremia or absence of detectable viremia following challenges (P = 0.01, for reduced early viremia, Mann-Whitney *U* test). A vaccine effect was statistically apparent, even if absence of viremia and delay of set-point viremia were treated as independent events (P < 0.02, combined test) which is highly unlikely to be the case as we saw increased immune responses towards vaccine antigens and novel responses towards the non-encoded gag sequence also in aviremic animals. Such detection of SIV protein specific responses strongly suggest occult infection, but the infection must have been kept at a very low level and/or abrogated early, as we used a very sensitive plasma based assay (Monjure et al., 2014) without detecting viremia for more than a year. Furthermore, we could not detect the viral DNA in lymph nodes in the aviremic animals using a highly sensitive PCR assay on sorted CD4 + T cells. Notably, such presumed T cell dependent early abrogation of detectable infection has previously been found in animals infected with live attenuated vaccines (Fukazawa et al. 2012; Hansen et al. 2011; Hansen et al. 2013).

Although our vaccine cannot have caused sterile immunity, the trend of 64% reduction of per-exposure risk of viremia we observed (P = 0.08) is comparable to that obtained using most other vectored systems that have targeted env in prime-boost regimens (Barouch et al. 2012; Barouch et al. 2015; Pegu et al. 2013; Selinger et al. 2014; Xiao et al. 2012). From a clinical perspective, prevention of viremia is a quite relevant readout and it would therefore be interesting to explore if it could also be observed in larger trials, or if it could provide an additive effect to other vaccine designs.

The mechanism of the profound control of acute viremia cannot be deduced from our observation as the animals responded rather homogenously with CD8 + T cells and not with CD4 + T cells, and we failed to recover responsive intestinal T cells pre-challenge for technical reasons. It is nevertheless worth noting that we previously observed CD8 + T cell dependent and MHC class II independent control of chronic viral infection (Holst et al., 2011), and that in the current study even the animals without directly measurable infection had recall tat, vif, rev and vpr vaccine primed T cell responses, gag specific T cell responses, and virus driven CD4 + T cell responses. These immune response patterns accurately mimics our observations during LCMV infection in inbred mice where occult infection was present, but undetectable using standard assays (Holst et al., 2015), and is similar to some of the effects observed during assumed preventive pharmacological inhibition of SHIV infection (Kersh et al. 2011; Promadej-Lanier et al. 2008; Tsegaye et al. 2015). However, unlike the LCMV studies in inbred mice where a subdominant antigen could reliably prevent exhaustion and allow the infection to raise dominant antigen specific T cells, this primate studies used randomly selected macaques. Notably, the two aviremic animals did express two of the three elite resistance associated alleles present in the vaccinated cohort (A*08 and B*17), and it is possible that this is part of the explanation of the observed effect. The absence of an early peak in virus replication that we observe in infected vaccines and indeed in animals that become infected and rapidly and durably control the infection is a highly unusual finding in vaccine studies. Other studies that successfully achieved partial control of replication even with multiple log reduction in peak viremia, still exhibit an early peak replication phase with normal viral kinetics (Casimiro et al. 2005; Liu et al. 2009). This normal consistency of peak viremia also includes the much larger Barouch et al. study which observed a combination of reduced acquisition and potent post-infection viremic control of SIV mac251 (Barouch et al., 2012). While the infection progresses different in our animals as compared to other vectored vaccination attempts, the course of infection in our infected vaccinees show some resemblance to the slow progression of infection in animals infected with an ultimately unsuccessful mismatched live-attenuated vaccine (Johnson et al. 1999; Wyand et al. 1999). This could be consistent with the original hypothesis rom the mouse studies, that viremic control is maintained by an infection primed response made possible by an early vaccine induced reduction in viral replication (Holst et al., 2015), with the caveat that SIV can gradually escape most T cell responses if near complete virological suppression is not achieved, but this could not be confirmed in our trial that was too small to perform meaningful correlate analysis. The task of achieving early reduction of virus control without dominant antigen was considered daunting from the onset and this is the reason why we used the MHC class II associated invariant chain adjuvant, a heterologous virus vectored prime-boost regimens and combined mucosal and parenteral immunization together with the unusual choice of antigens.

That the specific antigen choice or perhaps specific properties of the targeted antigens plays a unique role was suggested in a study by Hel et al., where a heterologous vectored virus vectored tat, rev and nef immunization was used and a small, but consistent delay in the time to reach peak viremia was reported (Hel et al. 2006). Immunizations with the tat antigen has also previously been claimed to reduce immune activation in patients on ART in prophylactic vaccination studies (Ensoli et al. 2010), and it is associated with protection against SHIV challenge in macaques (Bachler et al., 2013). However, in these cases protection was linked to antibody responses and we could not substantiate any association with tat antibodies and protection. Our vaccine did not include the dominant antigen nef and the early effects on early viral control were much greater in our study. Likely, the use of combined intramuscular and mucosal priming which allow mucosal effector cell recruitment to be maintained longer (Uddback et al., 2016), and/or the use of the MHC class II associated invariant chain as an adjuvant is responsible for the improved early control, but such causality is difficult to establish when using a repeated low-dose challenge regimen. Importantly for future studies attempting to build on our findings, the effects of the non-structural antigen vaccine in Hel et al. were additive with the structural gag-pol-nef vaccine also.(Hel et al., 2006). Thus, the ability to delay virus spread could possibly be applied to work in concert with T cells directed against other effective, but not normally very immunogenic epitopes targeting conserved regions of the HIV/SIV structural genes (Kulkarni et al. 2014; Mothe et al. 2015; Ondondo et al. 2016). When considering the future prospect of a partially effective vaccine, the absence of peak viremia is also an intriguing vaccine property in its own right. Thus, although the exact contribution of acute infection to forward transmission is unknown, epidemiological data have emerged to support a disproportionately large role, and genital mucosal secretions are considerably elevated in the weeks after acute infection (Miller et al. 2010; Pilcher et al. 2007; Volz et al. 2013). A vaccine that reduces acquisition as the RV144 trial or newer partly adenovirus based vaccines (Barouch et al., 2012; Barouch et al., 2015), as well as acute infectivity have an impact on further spread of the infection, and it would be predicted to synergize with treatment as a prevention strategy in reducing forward transmissions.

In addition to the effect on early virus replication, there were also signs in our study that the tvrv vaccine reduced the immune hyperactivation normally associated with HIV/SIV infection and disease progression (Fig. 5, Fig. 6). This is consistent with the virological findings as the immune scarring associated with acute infection must have been diminished, and it also holds clinical relevance. Thus, the HIV induced immune hyperactivation is a pathogenic consequence of infection that cannot be completely normalized with antiretroviral therapy (Cao et al. 2016; Hazenberg et al., 2003).

In summary, here we present a novel experimental vaccine regimen capable of inducing CD8 + T cell responses against subdominant antigens. This mucosally and systemically applied vaccine was capable of reducing early viral replication and reducing long term mucosal CD4 + T cell decline and immune hyperactivation, but it was not capable of inducing chronic controls except in those animals were durable control happened early and were we could never detect viremia. Our study demonstrates that responses targeting the SIV accessory antigens can effect early SIV replication and delay the chronic phase of the infection. In principle, such a vaccine capability could provide the time needed for durable effector mechanism to become active as it is seen during early treatment interrupted infection or live-attenuated vaccination. Clearly, in 4 out of 6 animals our vaccine and the infection combined could not elicit such responses in time, but it may be possible in future studies combining subdominant accessory antigen vaccines with other vaccines and antigens.

## Funding Sources

The study was funded by the Danish Research Council (0602-01464B), NIH grant R01 AI084793 (PI: R. Veazey) and the Danish AIDS-foundation (F10-18). Preliminary work was supported by the Hede Nielsen Family Foundation (2012-1176), the Foundation for the Advancement of Medical Sciences (90102), the Aase and Ejnar Danielsen Foundation (106740) and the Novo Nordisk Foundation (12678). The funding agencies had no influence on the execution of the project or its writing.

## Conflict of Interest Statement

The authors PJ Holst, JP Christensen and AR Thomsen are inventors on a manuscript related patent (published as WO2007062656). PJ Holst is a founder and shareholder of InProTher ApS, a biotech company which holds a license to use the invariant chain sequence for vaccination purposes against certain indication including HIV. The remaining authors have declared that no conflict of interest exists.

## Author Contributions

PJH conceived the project and wrote the first draft of the manuscript and all authors read and commented to the manuscript, HX, ER, DB and PJH performed experiments and analyzed data, AC, ER, BAHJ, ACM, PJH, SC, AF and RC, produced or contributed reagents, PJH, RSV, AN, JPC, JB, HX, AT and ART contributed to study design and discussions of results, and AT performed statistical analysis.

## Figures and Tables

**Table 1:** Tat antibody responses before and after challenge. The tat antibody responses were measured as end-point dilution titers in samples from individual macaques before the first challenge (90 days post booster immunization) and at the end of the trial (50 weeks post first challenge). Starting dilution was 1:200. A blank space denotes a titer of < 200.

Vaccination status	Monkey ID	Status	Pre-challenge	End of trial
Vaccinated	IT22	Protected		
	IA39	Partially protected		
	IT19	Protected		
	IH49	Partially protected		200
	IG98	Partially protected		400
	IJ60	Not protected	400	200
Not Vaccinated	IJ92	Not protected		
	II39	Not protected		
	IR98^a^	Not protected		
	IT06	Not protected		
	HV04	Not protected		
	IM44	Not protected		1600

a The indicated macaque had its last blood samples drawn 28 weeks after the first challenge.
